# Three-dimensional printing for cardiovascular diseases: from anatomical modeling to dynamic functionality

**DOI:** 10.1186/s12938-020-00822-y

**Published:** 2020-10-07

**Authors:** Hao Wang, Hongning Song, Yuanting Yang, Quan Cao, Yugang Hu, Jinling Chen, Juan Guo, Yijia Wang, Dan Jia, Sheng Cao, Qing Zhou

**Affiliations:** Department of Ultrasound Imaging, Renmin Hospital of Wuhan University, Wuhan, 430060 China

**Keywords:** 3D printing, Cardiovascular disease, Mock circulatory system

## Abstract

Three-dimensional (3D) printing is widely used in medicine. Most research remains focused on forming rigid anatomical models, but moving from static models to dynamic functionality could greatly aid preoperative surgical planning. This work reviews literature on dynamic 3D heart models made of flexible materials for use with a mock circulatory system. Such models allow simulation of surgical procedures under mock physiological conditions, and are; therefore, potentially very useful to clinical practice. For example, anatomical models of mitral regurgitation could provide a better display of lesion area, while dynamic 3D models could further simulate in vitro hemodynamics. Dynamic 3D models could also be used in setting standards for certain parameters for function evaluation, such as flow reserve fraction in coronary heart disease. As a bridge between medical image and clinical aid, 3D printing is now gradually changing the traditional pattern of diagnosis and treatment.

## Background

Three-dimensional (3D) printing is being increasingly applied to the treatment of cardiovascular diseases, especially in the diagnosis and treatment of structural heart disease [1–3]. Conventional, mostly rigid, 3D heart models are chiefly useful in displaying the cardiac structure. However, the development of flexible printing materials allows the fabrication of functional cardiovascular models that are further capable of hemodynamic testing and preoperative simulation. Their ability to help decide clinical strategy maximizes the value of 3D printing in this field.

### The origins and medical applications of 3D printing

Three-dimensional printing employs the principle of additive manufacturing to produce prototypes or final products by stereoscopically stacking discrete materials under computer control. Specific software slices a 3D digital model into several two-dimensional planes. Specific printing materials such as powder or resin are then accumulated layer-by-layer using a laser beam or hot melting. The superimposed layers then form the final product. The core idea of 3D printing originated at the end of the nineteenth century with the development of photosculpture and geomorphic forming technology. In 1984, Charles Hull applied optical technology to rapid prototyping, and in 1986 set up the world’s first company to produce 3D printing equipment, 3D Systems [4, 5]. Four core 3D-printing technologies—stereolithography (SLA) [6], selective laser sintering (SLS) [7], fused deposition modeling (FDM) [8], and three-dimensional printing (3DP) [9]—were patented between 1986 and 1993. They defined the preliminary development of the industry, and the current 3D printers still mainly employ them.

Recent medial applications of 3D printing include in dentistry [10–14], orthopedics [15–17], craniomaxillofacial surgery [18, 19], and even drug delivery [20–28]. For example, a 3D-printed SLS drill guide can be used to accurately sculpt a facial tumor [19]. Printed models can be used during orthopedic surgery to fix articular fractures and precisely position plates [16]. In drug delivery, digitally controlled 3D printing can create a personalized drug delivery system through the layer-by-layer fabrication of active and excipient ingredients according to the needs of the patient. Spritam®, containing the antiepileptic drug Levoacetam, is the first FDA-approved 3D-printed drug. When compared with traditional tablets, its pharmacological activity is similar, but the solubilization time is shorter.

**Fig. 1:**
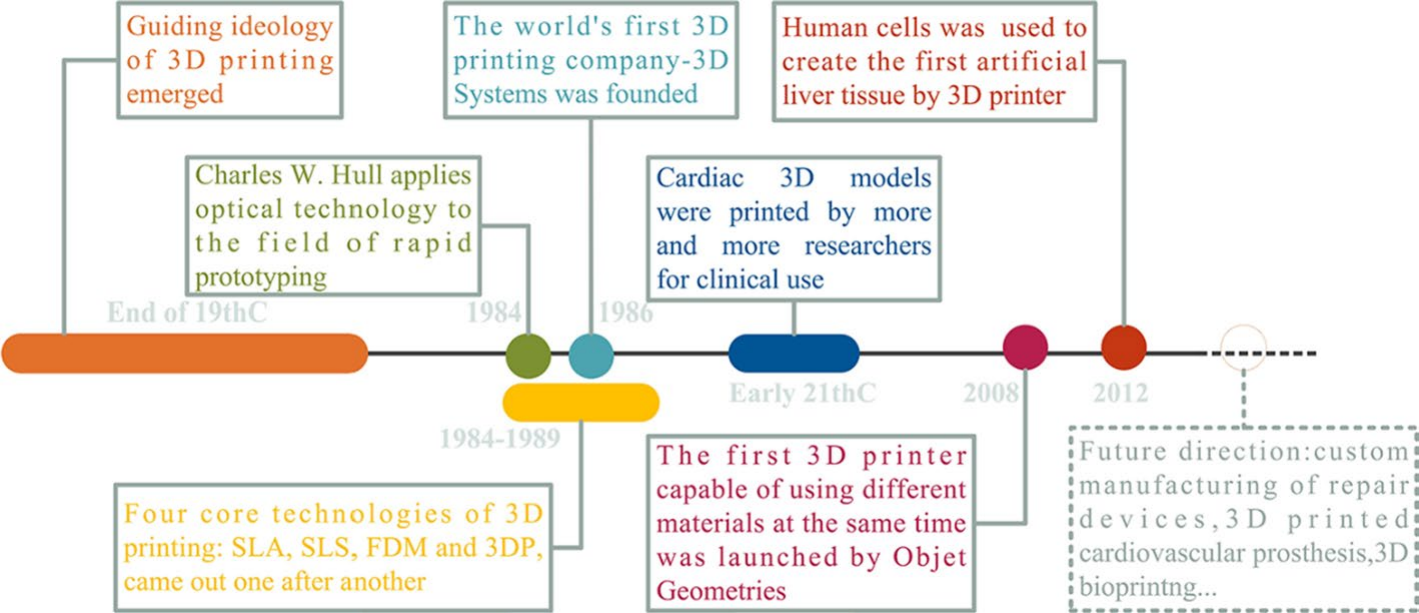
History of 3D printing and its medical applications

Although the research on applying 3D printing to the treatment of cardiovascular diseases is relatively scant, the technique can potentially play important roles in preoperative practice, diagnosis, and treatment. Schievano et al. [29] studied 12 patients who planned to undergo pulmonary valve implantation, and found that surgeons considered a 3D-printed heart model to be more beneficial than image data when determining whether to perform valve replacement. Sodian et al. [30] made a 3D model based on magnetic resonance imaging (MRI) data as an aid to determine an operation plan for a patient with a left subclavian artery complicated with descending aorta malformation. The disinfected model was then used in the operating room for intraoperative localization. Overall, printed cardiovascular models show great potential advantages in assisting both preoperative decision making and intraoperative navigation. The history of 3D printing and its medical applications are summarized in Fig. 1.

## Overview of cardiovascular 3D printing

### Imaging data acquisition

Accurate and reliable imaging data are the basis for accurate 3D model printing. At present, these data are most commonly from computed tomography (CT) imaging, cardiac MRI, and echocardiography. Cardiovascular CT imaging has high spatial and density resolutions, and so it is the most often used technique for cardiac modeling in clinical studies. As the heart is composed of muscle and connective tissue, its internal density differences are difficult to show clearly under the conditions of a plain CT scan. Angiography CT scanning can enhance the display of cardiac structures. In contrast mode, the areas of high-density display in CT images are cardiac chambers and blood vessels, so CT images are the preferred data source for 3D printing of the cardiac cavity and vascular structure [31–33]. Models for maxillofacial surgery usually use data reconstructed from slice thicknesses of 0.5–1 mm, whereas models of the pelvis and long bones can use slice thicknesses up to 2 mm. Cardiovascular modeling commonly employs 0.75 to 1 mm slice thicknesses with a smoother kernel. However, as CT scanning uses radioactive materials and is an invasive examination, not all cardiac structures are suitable for cardiac CT imaging. Cardiovascular magnetic resonance (CMR) has certain advantages over cardiac CT in displaying the heart’s soft tissue or valves [34–37]. Significant improvements to the spatial and temporal resolutions of transthoracic and transesophageal 3D echocardiography make it another alternative technology for acquiring reliable source data for 3D printing [38–41].

As the heart is a relatively complex organ, a single data source may not be able to provide a complete picture of its anatomy. The recent work has fused images from multiple sources for 3D printing [36, 42–44] to significantly improve the depiction of complex anatomical details in the heart and provide more comprehensive information to aid intervention for structural heart disease and preoperative planning.

### Imaging data postprocessing

Postprocessing isolates the target structures from a 3D volume dataset (in DICOM format) derived from medical imaging data. Commonly used software for medical image postprocessing includes 3D-Slicer, Horos, OsiriX, Mimics, and 3-Matic. These programs usually employ threshold segmentation to segment the required structure according to the different gray values of different tissues in the image. For example, the blood pool shows a lower signal intensity than the neighboring tissues, thereby delineating the intraluminal contour. The final digital model is exported in STL format and input into a 3D printer to obtain a 3D cardiovascular structure model.

### 3D printing procedures

As described above, 3D printers are of four main types: SLA, SLS, FDM, and 3DP. SLA uses liquid photosensitive resin for rapid prototyping, which can quickly condense to a solid under laser irradiation. Its major advantage is the ability to create complex shapes with internal structures and extremely high feature resolution (~ 1.2 μm) [45]. However, there are few types of photosensitive resin, and the cost is relatively expensive. FDM is the most widely used 3D printing method. It involves first melting the material, and then spraying it through a computer-controlled extrusion nozzle. A 3D model is established by the layer-by-layer accumulation of the material. Disadvantages include low printing accuracy and poor printing effect for soft materials. Detailed advantages and disadvantages of the four printing types are listed in Table 1.

**Table 1:** Comparison of four common 3D printing methods

Printing method	SLA	SLS	3DP	FDM
Resolution	High	Medium	Medium	Low
Surface quality	Smooth	Medium	Medium	Rough
Cost	Relatively expensive	Medium	Medium	Low-cost machines and materials
Materials	Photosensitive resin	Wax, metal, ceramic powder	Wax, metal, ceramic powder	Polymers: PLA, ABS, PVA
Other features	Limited to photopolymers; supports printing of flexible materials	Design freedom; no need for support; no post processing needed	Adhesive sprayed through nozzle	Limited to materials that melt

The financial and time costs depend greatly on the size and complexity of the printed structure and the material used. An FDM print of the mitral annulus takes about 30 min [46], whereas a simplified heart model may take about 3 days [47]. The photosensitive resin for SLA printing is usually more expensive than the plastic polymer used in FDM printing. An SLA print of the left atrial appendage using TangoPlus (a photosensitive resin) costs about $250 [48], whereas an FDM print of the full heart using ABS (a plastic polymer) only costs about $10 [49].

### Accuracy and reliability of 3D printed models

**Fig. 2:**
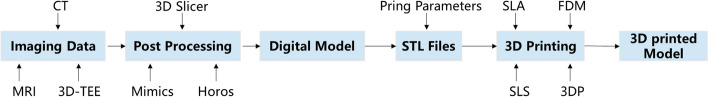
Workflow of 3D printing

The mean deviation between a 3D printed heart model and medical images is about 0.4 mm [50], which can be considered negligible in clinical use. This is because the 3D printing process works within tolerances, and models typically contract as the polymer solidifies. Greil et al. [34] used multislice CT and CMR imaging to scan the hearts of five patients with congenital heart disease, and obtained high-resolution 3D models by SLA printing, whose accuracy (about 0.15 mm) was higher than that of the imaging techniques, confirming that printing will not reduce the imaging accuracy. Figure 2 shows the workflow of 3D printing.

## Anatomical 3D models and cardiovascular disease

### Conventional materials used for 3D anatomical models

Conventional materials used for 3D printing (excepting bioactive materials used for 3D bio-printing) mainly include liquid photosensitive resin and polymers (either powders or fibers) [51]. In the past, 3D printing was mainly used to design personalized implants for orthopedic and joint surgery patients or to aid surgical navigation for such patients. Therefore, rigid materials, such as photosensitive resin and metal powder were mostly considered, as they meet the requirements of hardness and stress. More recent research has considered 3D printing polymers (such as PLA and ABS) for use in treating cardiovascular diseases. These materials are relatively inexpensive, and can depict specific structures of the heart, although with very different hardness and elasticity.

### Applications of anatomical 3D models in treating cardiovascular diseases

The above materials simulate only the shape but not the physical properties of cardiac and other tissue structures. They are therefore mostly used in the diagnosis and treatment of structural heart disease (such as cardiovascular disease and valvular disease), aneurysm, and other large vascular diseases, as they can clearly display pathological or abnormal structures. Models of congenital heart disease such as atrial and ventricular defects can clarify their locations and relationships with neighboring structures such as the superior and inferior vena cava, aorta, mitral valve, and tricuspid valve, and so they help to evaluate whether the patient is suitable for interventional closure and the selection of an appropriate size of occluder. Wang et al. [52] used CT data to print a 3D heart model of a patient after atrial septal defect surgery, which clearly showed that the sealing device completely covered the edge of the defect, and so confirmed the role of 3D printing in postoperative evaluation. Table 2 summarizes the cardiovascular applications of 3D models.

**Table 2:** Applications of 3D anatomical models to treating cardiovascular diseases

Condition	Material	Printing method	Purpose
Congenital heart disease			
Atrial septal defect [33, 53, 54]	PLA, resin, polyurethane filament	FDM SLA	Preoperative evaluation; transcatheter device closure simulation
Ventricular septal defect [55–58]	PLA, gypsum, cyanoacrylate	FDM	Congenital heart disease education for medical students; transcatheter device closure simulation
Complex congenital heart disease: e.g., endocardial cushion defect [54, 59], double-outlet right ventricle [56, 60–62]	PLA, resin, VeroMagenta	FDM SLA	Improve understanding of congenital heart disease; surgical management
Heart valve disease			
Mitral valve disease [63–65]	PLA, ABS	FDM	Surgical management
Tricuspid valve disease [66]	ABS	FDM	Clinical decision-making; surgical planning; education
Aortic valve disease [67, 68]	Resin, PLA	FDM SLA	Surgical planning and training
Others			
Arterial aneurysm [69]	Resin	SLA	Preoperative planning; postoperative evaluation

### Limitations of anatomical 3D cardiovascular models

Although anatomical models can assist clinicians, especially surgeons, in planning operations and selecting devices, it is not guaranteed that a decision made using a rigid 3D model perfectly suits in vivo reality. Consider an atrial septum defect, for which the compression ratio of an implanted occluder and the shunt around it under normal cardiac pressure could clearly not be obtained using a rigid 3D model with very different properties form the actual heart. Researchers have therefore attempted to maximize the advantages offered by 3D printing by seeking realistically functioning cardiovascular models (with such as opening and closing valves and pulsating blood vessels). The recent emergence of flexible 3D printing materials (like TangoPlus) and the establishment of mock circulatory systems in vitro represent significant progress. Relevant research has combined 3D printing with mock circulatory systems to produce functional models of blood vessels, valves, and other structures to maximize the clinical usefulness of 3D printing.

## Functional models and cardiovascular disease

**Fig. 3:**
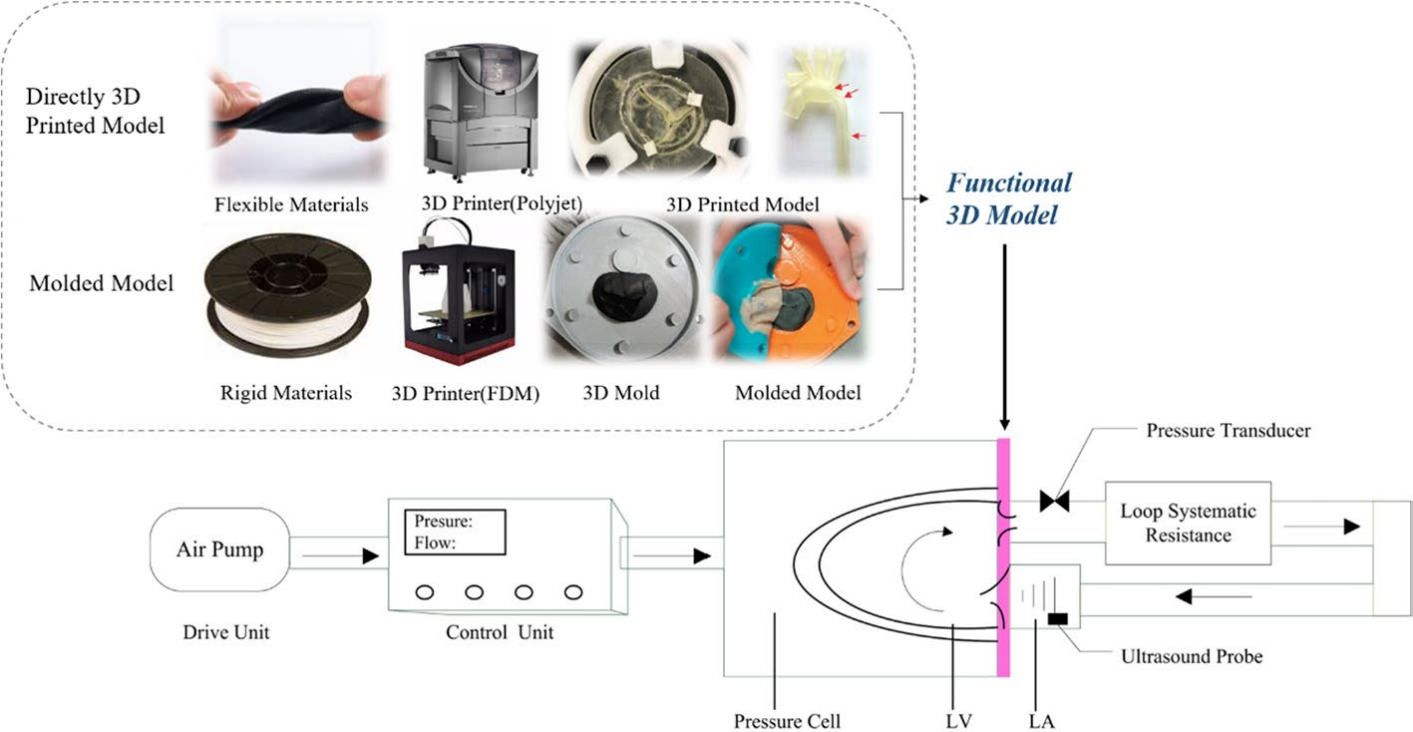
Functional 3D models and their coupling with a mock circulatory system. Images in the figure are from the literature [72–74]

To properly simulate tissue function requires both a flexible 3D cardiovascular model and its coupling with a mock circulatory system. Functional 3D models and their coupling with a mock circulatory system are shown in Fig. 3.

### Flexible materials for 3D cardiovascular models

Any model for studying hemodynamics must accurately replicate the mechanical properties of the relevant tissues, and so must be more elastic and softer than conventional 3D printing materials. Recent advances include the 3D printing of rubber-like materials, which are gradually becoming widespread. A representative example is the TangoPlus series of materials, which can be printed as soft heart valves. The Connex3 Ojet series of 3D printers (Stratasys, Eden Prairie, MN, USA) can combine materials of different color and hardness, allowing reproduction of complex cardiac structures. Vukicevic et al. [70] used TangoPlus and Verowhite, respectively, to simulate the mitral valve and calcification on it, and tested the resulting model’s mechanical parameters (such as bending and tensile moduli) in comparison with porcine leaflet tissue; the parameters were close to and consistent with those of the tissue. Maragiannis et al. [71] used the same two materials to create aortic valve complexes, and confirmed their feasibility for ultrasound imaging. Biglino et al. [72] made a blood vessel model using TangoPlus, and demonstrated its suitability for fabricating arteries, although its mechanical properties might not be suitable for modeling softer blood vessels.

Although directly printing rubber-like materials, such as TangoPlus can largely simulate the mechanical properties of cardiovascular tissues, it is relatively expensive. Using a rigid 3D model as a mold, a flexible model can alternatively be obtained by painting it with silicone (such as smooth-on, Echoflex). Scanlan et al. [73] compared 3D models of the tricuspid valve obtained by the two methods, and reported that both closely replicated the patient’s anatomy, while the silicone model was better at simulating the texture of cutting and stitching. Based on the ultrasound images, a mitral valve prolapse model created by Olivia et al. [74] using Echoflex 00–30 also had good fidelity to the preoperative three-dimensional echocardiogram. Such materials are promising for the production of low-cost functional 3D cardiovascular models. The properties of common materials used in 3D printing are compared with those of human tissues in Table 3.

**Table 3:** Properties of common materials used in 3D printing compared with those of human tissues

Material	Printing method	Elastic modulus (MPa)	Shore hardness	Cost
Printing material^a^				
PLA	FDM	3000–4000	–	Low
ABS	FDM	2200	–	Low
Verowhite	SLA (Polyjet)	2000–3000	83–86 Scale D	Medium
VeroClear	SLA (Polyjet)	2000–3000	83–86 Scale D	Medium
Mold Star 15	Casting on 3D printed model	2.7	15 Scale A	Low
Echoflex 00–30	Casting on 3D printed model	1.4	00–30	Low
TangoPlus FLX930	SLA (Polyjet)	–	26–28 Scale A	Expensive
Human Body				
Aorta/vena cava	–	0.04–1.9 [75]	–	–
Valves	–	1.0–1.3 [76]	–	–
Vasculature	–	0.1–0.55 [76]	–	–
Heart muscle	–	0.08 [76]	–	–

^a^Data from manufacturers’ websites

00–30: Grade 00 hardness, much lower than shore hardness

### In vitro mock circulatory systems

A mock circulatory system is an experimental simulation of human hemodynamics that can be used to evaluate ventricular assistance devices, artificial valves, and other artificial cardiovascular components and to study the blood flow characteristics of cardiovascular diseases. Its basic components are described below.

#### Drive unit

A vacuum pump or air compressor is usually used as an actuator to power the device. The actuator is externally connected to a programmable logic controller to control the pulse of the heart or blood vessels. Pantalos et al. [77] placed a silicone ventricle in a chamber full of compressed gas to control its rhythmic contraction and relaxation. Olivia et al. [74] used compressed gas to drive pneumatic pistons, which were connected to contractile loops around different levels of a ventricle model, to simulate the movement of different ventricular segments.

#### Blood flow test system

*Doppler ultrasound*: Doppler ultrasound can display the morphological characteristics of cardiovascular structures, evaluate the diameters of heart chambers and blood vessels, and monitor the blood flow velocity and pressure at different observation points in real time. Doppler flow imaging can also be used to evaluate the effects of surgery simulated in vitro, such as whether there is perivalvular leakage after valve replacement and whether there is residual shunt around the device.

*4D-flow MRI: *With the development of MRI technology and related software, 4D-flow MRI is gradually being applied in scientific research and clinical practice. It is a phase contrast technique that codes flow rates in the *x*-, *y*-, and *z*-directions [78]. It can quantitatively and visually evaluate characteristics of flow velocity (e.g., minimum and peak velocity, stroke volume, net flow, and reverse stroke) and wall shear stress (e.g., flow rate, pressure differential diagram, pulse wave velocity, and energy loss). The technique’s disadvantages include long imaging times and the time-consuming postprocessing analysis.

*Catheter-based monitoring: *With a monitoring system such as Mac-Lab, a catheter-based monitoring device can plot pressure with respect to time. The plot’s peak (rising or falling branch) can be used to evaluate fluid parameters such as vascular resistance and flow rate.

*Particle image velocity measurement*: Particle image velocity measurement is an optical method of measuring flow velocity by capturing (and analyzing) multiple snapshots to record the positions of particles in the flow field. It is commonly used for fluid imaging, and can easily obtain physical information, such as eddy currents and pressures, but it has high optical requirements for imaging particles in the fluid [79].

#### Blood mimicking fluid

Simulations of blood flow resistance generally use solutions, such as mixtures of water and glycerin that have a viscosity similar to that of blood (3.5–4.5 mPa /s). However, different test systems have different requirements for blood mimicking fluids. When using Doppler ultrasound, the addition of 5 μm diameter nylon scattering particles to a fluid matrix containing water, glycerol, dextran, and surfactants improves the acoustic properties of the fluid and provides better imaging [80]. Velocity measurement using particle images requires the addition of tracer particles to the fluid.

### Application of functional cardiovascular models in diagnosis and treatment

#### Heart valve disease

Heart valve disease mainly includes valve stenosis and regurgitation, and usually involves changes in the structure and/or function of a valve owing to various causes. Recent advances in interventional operation, transcatheter valve replacement, valve repair, and valvuloplasty have increased treatment options. Accurate assessment of the location and extent of heart valve disease before surgery is therefore becoming increasingly important to treatment. Conventional heart valve models can display anatomical features, but cannot test their function. Printing a functional valve model with flexible materials and placing it in a mock circulatory system to test velocity, pressure gradient, and other parameters can help understand the severity of valvular disease. It can also simulate the operation to aid device selection, predict possible complications, and improve its success rate.

*Severity assessment and surgical simulation of valvular disease*: Maragiannis et al. [71] used TangoPlus and Verowhite to simulate the aortic valve leaflet and valvular calcification, respectively. They printed valve models of eight patients with severe aortic stenosis, and placed them in a mock circulatory system. Valve orifice area was measured by ultrasound Doppler and Gorlin formula to evaluate the severity of the disease. The blood flow velocity, pressure gradient, and valve orifice area measured on the model were consistent with in vivo measurements. This confirms the feasibility of using a 3D-printed functional aortic valve model combined with a simulated circulatory system to assess the severity of valve stenosis.

Vannelli et al. [81] created a left heart system model including left ventricle, aortic valve, and mitral valve by mold modeling. Driven by gas pressure, it simulated the contraction and relaxation of normal human ventricles and the opening and closing of the valve, with a left ventricular ejection fraction (51%) within the normal range. Azad Mashari et al. [82] used similar devices to simulate the motion state of a pathological mitral valve during opening and closing, and obtained a blood flow spectrum consistent with that measured in vivo. Although the above studies to a certain extent simulated the motion of normal and pathological mitral valves and in vivo hemodynamic characteristics, due to the lack of systematic resistance in the device, the anterior velocity and pressure gradient generated by the ventricle during systole were not sufficient, and the results were not satisfactory. Olivia et al. [74] created 3D models of 10 patients with mitral valve regurgitation, and set up a pipe about 0.85 m high in a mock circulatory system to simulate the hydrostatic pressure generated by the aorta in vivo. The device could generate 107 mmHg pressure during systole, more accurately reflecting the in vivo hemodynamic state. In addition, this study also simulated mitral valve repair, mitral clip, and other operations in 3D models, and determined the best surgical strategy by observing the changes in hemodynamics before and after surgery. It evaluated the effects of surgery, and predicted possible postoperative complications. Ultrasound images collected from the model were consistent with in vivo images, confirming the clinical value of 3D-printed heart valves in the diagnosis and treatment of diseases when coupled with mock circulatory devices.

*Hemodynamic testing of artificial valves*: In the past decade, transcatheter aortic valve replacement has become a life-saving alternative for patients who cannot tolerate conventional valve replacement [83]. The hemodynamic assessment of prosthetic valves relies on 3D-printed cardiovascular models to reproduce the anatomical structure and a mock circulatory system to simulate the in vivo blood flow state. Wentao Feng et al. [84] explored the possible deformation of artificial valve devices with different leaflet thicknesses for a patient with aortic root calcification by evaluating the effective valve area of the prosthetic valves, the average transvalvular pressure, and the valvular regurgitation. As the thickness of the valve increased, valve regurgitation due to device deformation became more severe. De Gaetano et al. [85] tested the pressure gradient, flow rate, effective valve orifice area, reverse flow, and other parameters of a new polymer heart valve under continuous and pulsating flow conditions using a mock circulatory device, and expected that this new kind of valve would soon find clinical applications. The above studies prove that 3D printing coupled with a mock circulatory system can help evaluate artificial valves and develop new devices for clinical use.

#### Coronary heart disease

Coronary artery disease, usually caused by coronary artery atherosclerosis, is one of the world’s most fatal diseases. Percutaneous coronary intervention, especially intracoronary stent implantation, has proven to be an effective treatment. However, postoperative stent restenosis is a persistent problem. Improper stent implantation positions will enlarge the area of low WSS and subsequently stimulate epithelial cells in the vascular wall to grow outward; thereby, increasing the risk of postoperative restenosis [86]. HuJun Wang et al. [87] investigated the effects of different stent implantation positions on coronary artery hemodynamics and the area of the low-WSS region using 3D coronary artery models. The initial strategy was ostial stenting with the stent’s top end staying in the upper area of the original stenosis. Three subsequent tests each raised the stent position 1.38 mm upward along the branch. The second position, called the half-cover strategy, had the fewest low-WSS areas, and was considered the best among the four compared. This shows that 3D printing coupled with a mock circulatory system can find the best position for implanting a coronary stent.

In addition to clinical strategy planning, 3D printing coupled with a mock circulatory system can also be used for setting standards for certain parameters for function evaluation. In addition to coronary angiography, intravascular ultrasound, and optical coherence tomography, the coronary flow reserve fraction (FFR), a function evaluation parameter for coronary artery stenosis and ischemic assessment, has received increasing attention. It is defined as the ratio of mean intravascular pressure in the distal end of coronary lesions to that in their proximal end in the state of maximal myocardial microcirculation congestion induced by adenosine and other drugs [88]. Kranthi et al. [89] used a 3D coronary model to test its variation in coronary stenosis vessels under different aortic pressures, finding that for a given stenotic vessel, the value gradually decreased as the aortic pressure increased. This also provides a reference for setting a standard of FFR for the evaluation of coronary stenosis in vivo.

#### Vascular disease

The treatment and diagnosis of aneurysms and macrovascular diseases can be aided using 3D-printed models coupling with a mock circulatory system. Anderson et al. [90] combined 3D printing with 4D phase contrast MRI in the visualization and quantification of blood flow characteristics in aneurysms. Hemodynamic simulation could also assess the rupture risk of aneurysm in vitro. In addition, the study also simulated the implant of a diverter in vitro, and observed changes of local hemodynamic characteristics before and after surgery, which helped predict the outcome of the procedure. Biglino et al. [72] used TangoPlus for 3D modeling and in vitro hemodynamic testing of patients with left ventricular hypoplasia syndrome with aortic coarctation. The anatomical structures of the ascending aorta, aortic arch, and descending aorta of the patient were reconstructed. The model did well in replicating capacity bearing (70/40 mmHg), material compliance under pressure, and the consistency and anatomical structure. Knoops [91] used latex to make an adult lung circulation model. Wave intensity analysis identified three main waves (forward compression, backward compression, and forward expansion) in the main pulmonary artery. The model can be applied to the diagnosis of pulmonary hypertension, pulmonary unilateral stenosis, abnormal blood flow shunt after repair of transposition of the great arteries, and pulmonary artery disease such as Eisenmenger syndrome.

Table 4 summarizes applications of functional 3D models in treating cardiovascular diseases.

**Table 4:** Applications of functional 3D models in treating cardiovascular diseases

Application	Materials	Purpose
Heart valve condition		
Aortic valve stenosis [71]	TangoPlus, Verowhite	In vitro assessment of stenosis severity
Mitral valve stenosis [82]	Mold star 15, Ecoflex 00–30	In vitro assessment of stenosis severity
Mitral valve regurgitation [74]	Mold star 15, Ecoflex 00–30	Surgical simulation: mitral valve repair, mitral-clip
Artificial valve [84, 85]	Silicone	Device development: exploring relationships between artificial valve thickness and valve function
Coronary heart disease		
Coronary heart disease [87, 89]	Wax, VeroClear	Surgical planning: optimal stent placement; parameter evaluation criteria: FFR
Vascular disease		
Intracranial aneurysm [90]	Semi-translucent PLA	Surgical simulation: diverter implantation
Abdominal aortic aneurysm [92]	Polyjet Material Rubber FLX930	Surgical simulation: transcatheter intervention repair

## Limitations

A variety of factors, including excitation of autonomic nerves and endocrine regulation, affect hemodynamic characteristics. Although 3D printing can accurately reproduce the anatomical structures of the heart and the intracardiac pressure, it cannot simulate the in vivo physiological environment. Any parameters obtained using a simulation only reflect the intracardiac hemodynamic characteristics to a certain extent, which the model will not completely match. A further limitation is the enduring high cost (in terms of both time and money) of building an accurate dynamic 3D model. Furthermore, the potential clinical value of printed models, beyond “viewing” and preoperative simulation, needs to be further explored.

## Future Prospects

Developments of 3D printing technology have attracted increasing numbers of researchers to apply it to biomedical engineering, especially in tissue engineering and regenerative medicine. When compared with traditional tissue engineering, which first prints a scaffold and then inoculates it with cells, 3D bioprinting could directly print different types of cells in the right spatial locations. By seeding cells into a 3D tissue-like structure, this technology could obtain higher cell density and realize a uniform cell density distribution inside and outside the tissue. Direct 3D bioprinting of tissue or organs containing living cells has shown great potential, and has been widely studied, with examples including heart valves [93–95], skin [96, 97], nerves [98], liver tissue [99], alveoli [100], and even corneas [101] all being reported. More research is needed to meet the existing challenges of such as vascularization, organ rejection, and the lack of ideal bio ink.

Computational fluid dynamics (CFD) is another field receiving much research interest. CFD programs include ANSYS (ANSYS Inc., Pittsburgh, America) and COMSOL Multiphysics (COMSOL Inc., Stockholm, Sweden). Digital 3D modeling can be imported into CFD software after meshing. After setting boundary conditions, hemodynamic parameters at any point inside the model are easily obtained by CFD. CFD simulation has been used in drug delivery [102, 103] and the development of medical devices [104]. Pourmehran et al. [102] used CFD to simulate the air flow and magnetic particle deposition in a realistic human airway geometry obtained from CT images, thereby improving the efficiency of targeted drug delivery to the human lung in the presence of an external nonuniform magnetic field. CFD has also shown strong advantages in disease modeling and drug delivery.

Computer-aided design (CAD) was also an alternative method in planning interventions/treatment of cardiovascular diseases. For example, transcranial mitral valve replacement (TMVR) is an emerging method for the treatment of patients with severe mitral valve disease. However, the left ventricular outflow tract (LVOT) obstruction was a potentially fatal complication after surgery. Sung-Han Yoon et al. [105] successfully used CAD model based on the CT dataset to predict new LVOT area; thereby, prevent possible adverse clinical outcome. Besides, augmented reality technique has gained more attention and help plan surgery too [106].

## Conclusion

There are wide ranging medical uses of 3D printing technology, but most research still focuses on anatomical models. The transition from static modeling to dynamic functionality requires more research and the development of materials closer in properties to human cardiovascular tissue. The combination of 3D-printed cardiovascular models and mock circulatory systems also depends on cross-disciplinary cooperation in biology, electrical engineering, and computer science. Further technological developments will ensure the increasingly important roles of 3D-printed functional heart models, maximizing the value of 3D printing in the treatment of cardiovascular diseases.

## Data Availability

The authors will make all data detailed in this paper freely available.

## Abbreviations

MCS: Mock Circulatory System
SLA: Stereo Lithography Appearance
SLS: Selective Laser Sintering
FDM: Fused Deposition Modeling
3DP: Three-Dimensional Printing
PLA: Polylactic Acid
ABS: Acrylonitrile Butadiene Styren
